# Application of Long Sequence Reads To Improve Genomes for *Clostridium thermocellum* AD2, *Clostridium thermocellum* LQRI, and *Pelosinus fermentans* R7

**DOI:** 10.1128/genomeA.01043-16

**Published:** 2016-09-29

**Authors:** Sagar M. Utturkar, Edward A. Bayer, Ilya Borovok, Raphael Lamed, Richard A. Hurt, Miriam L. Land, Dawn M. Klingeman, Dwayne Elias, Jizhong Zhou, Marcel Huntemann, Alicia Clum, Manoj Pillay, Krishnaveni Palaniappan, Neha Varghese, Natalia Mikhailova, Dimitrios Stamatis, T. B. K. Reddy, Chew Yee Ngan, Chris Daum, Nicole Shapiro, Victor Markowitz, Natalia Ivanova, Nikos Kyrpides, Tanja Woyke, Steven D. Brown

**Affiliations:** a Graduate School of Genome Science and Technology, University of Tennessee, Knoxville, Tennessee, USA; b Department of Biological Chemistry, The Weizmann Institute of Science, Rehovot, Israel; c Department of Molecular Microbiology and Biotechnology, Tel-Aviv University, Ramat Aviv, Israel; d Biosciences Division, Oak Ridge National Laboratory, Oak Ridge, Tennessee, USA; e BioEnergy Science Center, Oak Ridge, Tennessee, USA; f Institute for Environmental Genomics, University of Oklahoma, Norman, Oklahoma, USA; g DOE Joint Genome Institute, Walnut Creek, California, USA

## Abstract

We and others have shown the utility of long sequence reads to improve genome assembly quality. In this study, we generated PacBio DNA sequence data to improve the assemblies of draft genomes for *Clostridium thermocellum* AD2, *Clostridium thermocellum* LQRI, and *Pelosinus fermentans* R7.

## GENOME ANNOUNCEMENT

Draft genome sequences have been generated for *Clostridium thermocellum* ad 2 (1), *Clostridium thermocellum* LQRI (DSM 2360) (2), and *Pelosinus fermentans* R7 (3), which encompassed 131, 110, and 65 contigs, respectively.

*Clostridium* (*Ruminiclostridium*) *thermocellum* strains are known for their potent cellulolytic capabilities (4). *C. thermocellum* strain AD2 is derived from a cellulose adhesion-defective (AD) mutant strain and played a critical role in describing the original cellulosome concept (5). *C. thermocellum* strain LQRI is an LQ8 reisolate used in enzyme studies to propose electron flow paths (6), and has been mistakenly called LQR1 on occasion. A genome for LQ8 (DSM 1313) has been reported (7). *P. fermentans* type strain R7 belongs to the *Negativicutes* within the *Firmicutes* phylum and in the presence of a fermentable substrate it can reduce Fe(III) (8). Complete genomes for *Pelosinus fermentans* JBW45 (9) and *Pelosinus* sp. strain UFO1 (10) have recently been reported using only single-molecule DNA sequencing technology, and the utility of long read sequences has been shown to improve other microbial genome assemblies (11–15).

In this study, genomic DNA of all three strains underwent Pacific Biosciences RSII standard template preparation and sequencing. Raw reads were assembled using the HGAP (version: 2.3.0) protocol. The HGAP assembly of AD2 contained 10 contigs and the 3.55-Mb finished genome was generated by superassembly using the Geneious (version 8.1.6) software combined with PCR and Sanger, as described previously (14). The HGAP assembly for LQRI contained two contigs, totaling 3.61 Mbp in size, with an input read coverage of 166.7. A small duplicated 12-kb LQRI artifact contig was removed from the assembly, leaving a circular chromosome of 3.57 Mb. The final HGAP assembly for the R7 genome contained two contigs totaling 5.02 Mb in genome size. Genes for all three genomes were identified using Prodigal (16) and annotations were performed as described previously (17). A total of 3,055, 3,071, and 4,631 protein-coding genes were identified in the AD2, LQRI, and R7 genomes, respectively.

A prior comparison of single-molecule sequencing-based genome assembly to short-read and hybrid assembly approaches showed that assemblies based on shorter-read technologies were confounded by a large number of longer repeats, in particular multiple copies of ~5-kb rRNA gene operons (11). Consistent with this observation, earlier drafts of AD2, LQRI, and R7 genome sequences contained single copies of the rRNA genes. The improved genomes for the *C. thermocellum* strains reported in this study each contain four copies of the 5S, 16S, and 23S rRNA genes. The improved *P. fermentans* R7 genome reported in this study contains 12, nine, and nine copies of the 5S, 16S, and 23S rRNA genes, respectively.

Finished or near-finished genome assemblies for these strains represent a significant improvement over earlier draft assemblies. We expect that the protein-coding potential will be superior (14) and that these genomes will facilitate comparative and functional genomic studies. Lastly, these new genome sequences will also facilitate a broader and more detailed comparison between sequencing and assembly technologies.

### Accession number(s).

This whole-genome shotgun project has been deposited at DDBJ/ENA/GenBank under accession numbers CP013828, CP016502, and AKVN00000000 for AD2, LQRI, and R7, respectively.
